# Anthocyanin-rich extract from purple tea: Chemical stability, cellular antioxidant activity, and protection of human erythrocytes and plasma

**DOI:** 10.1016/j.crfs.2024.100701

**Published:** 2024-02-16

**Authors:** Cristiane de Moura, Mariana Araújo Vieira do Carmo, Yong-Quan Xu, Luciana Azevedo, Daniel Granato

**Affiliations:** a Department of Chemistry, State University of Ponta Grossa (UEPG), Av. Carlos Cavalcanti, 4748, 84030-900, Ponta Grossa, Brazil; b LANTIN – Laboratory of Nutritional and Toxicological Analyses in vitro and in vivo, Federal University of Alfenas (UNIFAL-MG), Rua Gabriel Monteiro da Silva, 714, 37130-000, Alfenas, Brazil; c Tea Research Institute, Chinese Academy of Agricultural Sciences, Key Laboratory of Tea Biology and Resources Utilization, Ministry of Agriculture and Rural Affairs, 9 South Meiling Road, Hangzhou, 310008, China; d Bioactivity & Applications Lab, Department of Biological Sciences, Faculty of Science and Engineering, University of Limerick, V94 T9PX, Limerick, Ireland

**Keywords:** Phenolic compounds, Functional foods, Bioactivity, Natural colourants, Cell culture, Oxidative stress

## Abstract

This study aimed to obtain an anthocyanin extract from the purple leaves of *Camellia sinensis* cv. Zijuan using a sustainable, non-toxic, and low-cost solid-liquid extraction, employing an aqueous citric acid solution (0.2 mol/L) as the extracting solvent, and to evaluate its chemical stability at different pH values, as well as its *in vitro* antioxidant properties in chemical and biological terms. The phenolic composition, *in vitro* antioxidant activity, and the stability of anthocyanins against pH, temperature, and light of the crude extract (CE) were evaluated, as well as the phenolic composition and bioactivity in the crude lyophilised extract (CLE). In the direct/reverse spectrophotometric titration, anthocyanins showed structural changes between pH 2 and 10, and reversibility of 80%. The antioxidant activity against the DPPH radical showed inhibition percentages of 73% (pH 4.5) to 39% (pH 10). Thermal stability was observed at 60 °C, and prolonged exposure of the extract to light caused photodegradation of the anthocyanins. Thirty-three phenolic compounds, including anthocyanins and catechins, were quantified in the CLE by UPLC-ESI-MS and HPLC, totalling 40.18 mg/g. CLE reduced cell viability (IC_50_ from 18.1 to 52.5 μg GAE/mL), exerted antiproliferative (GI_50_ from 0.0006 to 17.0 μg GAE/mL) and cytotoxic (LC_50_ from 33.2 to 89.9 μg GAE/mL) effects against A549 (human lung adenocarcinoma epithelial cells), HepG2 (hepatocellular carcinoma), HCT8 (ileocecal colorectal adenocarcinoma), and Eahy926 (somatic cell hybrid cells); and showed protection against oxidation of human plasma (635 ± 30 mg AAE/g). The results showed the diversity of compounds in the extracts and their potential for technological applications; however, temperature, pH, and light must be considered to avoid diminishing their bioactivity.

## Highlights

• The crude extract was obtained low-cost and non-toxic method.
• Diverse phenolic composition and antioxidant properties was observed.
• Thermal stability of extract was observed at 60 °C.
• The extract showed protection against oxidation of human plasma.
• The results showed their potential for technological applications.

## Graphical abstract

**Figure:**
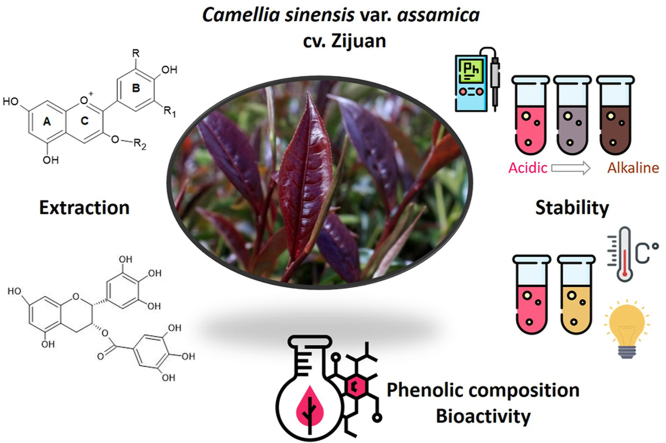

## 1 Introduction

Popularly known as purple tea, Zijuan tea is a cultivar of *Camellia sinensis* var. *assamica* [*Camellia sinensis* (L.) var. *assamica* (Masters) Kitamura] with purple leaves (Lv et al., 2015; de Moura et al., 2022). This colour comes from the accumulation of anthocyanins; exposure to adversity, such as intense light in summer or low temperatures in autumn, causes the purple colour to prevail even after favourable environmental conditions are restored. (Li et al., 2023). Its chemical composition also contains caffeine and flavan-3-ols such as (−)-epigallocatechin gallate (EGCG) (Kerio et al., 2013; Lv et al., 2015; Shen et al., 2018; de Moura et al., 2023). Purple tea extracts have demonstrated biological effects (Fan et al., 2013; Kerio et al., 2013; Gao et al., 2018; Zhao et al., 2020), and provide potential new applications for the plant as a functional ingredient.

It is common to use antioxidants in foods to increase the durability and quality of products during storage and use, interrupting oxidation processes, caused by reactive oxygen species (ROS), which can attack and damage molecules, such radicals attack through the donation of unpaired electrons or capturing electrons from another molecule, remaining stable, but the attacked molecule transforms into a radical, triggering a chain reaction. Examples of ROS include superoxide anion (O_2_^•-^), hydroxyl radical (^•^OH), nitric oxide (NO), and hydrogen peroxide (H_2_O_2_), which, despite not being a free radical, can cause irreversible injuries to the human cell membranes (Zenin et al., 2022). It should be noted that ROS are responsible not only for reducing the quality of the product but also for negatively impacting the human body, causing many diseases, and accelerating the ageing process.

Consumer concerns about health have resulted in the increased extraction of phenolic compounds from natural sources and their use as food colourings and preservatives to replace synthetic alternatives. The use of crude extracts or isolated compounds as additives by the food industry makes it possible to develop innovative, healthy, nutritious products with a long shelf life, thereby attracting consumers' attention and boosting sales of these foods (Granato et al., 2018; Gonçalves Bortolini et al., 2021). Among the compounds listed as GRAS (Generally Recognized as Safe), anthocyanins are the main compounds of interest due to the palette of colours they can produce, from red to blue and purple (Khoo et al., 2017; Enaru et al., 2021). However, crude extracts made from plant material have low efficiency; their yield is usually less than 1% of dry matter. Therefore, the extraction approach must provide a high phenolic yield and use safe solvents for consumers and the environment (de Moura et al., 2023). The use of mathematical models obtained by the response surface methodology (RSM) aids the understanding of the effect of different factors (e.g., extraction time and temperature) on the chemical composition and bioactivity of plant-based extracts (Armstrong et al., 2020; Escher et al., 2020) and assists in the choice of ideal parameters for extraction (de Moura et al., 2022), favouring better efficiency and yield.

Since 2012, there have been reports of identifying anthocyanins and anthocyanidins in extracts from Zijuan purple tea. Kerio et al. (2012) identified five of the six most common natural anthocyanidins in purified methanolic extracts of fermented (*black tea*) and unfermented (*green tea*) Zijuan tea, namely delphinidin, cyanidin, pelargonidin, peonidin and malvidin. More recently, in a study by Chen et al. (2023), the authors evaluated different samples of Zijuan tea from regions in China (Yunnan province, Qijiang and Ersheng district) and found the presence of 34 anthocyanins in the methanolic extract acidified with hydrochloric acid of the teas. Of these, peonidin-3*-O-*(6-*O*-*p*-coumaroyl)-glucoside, pelargonidin-3-*O*-galactoside, cyanidin-3-*O-*galactoside, cyanidin-3-*O-*(6-*O*-*p*-coumaroyl)-glucoside, delphinidin-3-*O-*(6-*O*-*p*-coumaroyl)-glucoside, and delphinidin-3-*O*-galactoside were found in the highest concentrations.

The chemical structure of anthocyanins attributes various biological activities to the molecules, such as antioxidant and anti-inflammatory activity, regulating apoptosis, participating in enzyme activation, cellular interactions, signal induction, and receptor activation (Gao et al., 2018; Migliorini et al., 2019; Alappat and Alappat, 2020). In acidic solutions (pH between 1 and 2), anthocyanins are more stable since they exist primarily as a flavyl cation (AH^+^). In contrast, pH levels above 6 can cause the heterocyclic ring to break, making the reaction irreversible and losing biological activity (Migliorini et al., 2019; Enaru et al., 2021). High temperatures increase the rate of thermal degradation of anthocyanins, in addition to exposure to light, which results in colour changes due to the formation of brown degradation products (Pedro et al., 2016a, Pedro et al., 2016b; Escher et al., 2020).

There is no previously published research about obtaining an extract from purple tea rich in anthocyanins, coupled with evaluating its chemical stability and antioxidant potential in different biological media. Thus, this study aimed to obtain an anthocyanin-rich extract from the purple leaves of *C. sinensis* (Zijuan Tea), obtained using non-toxic, food-standard solvents, and to evaluate its chemical stability at different pH values, as well as its *in vitro* antioxidant properties in chemical and biological terms.

## 2 Materials and methods

### 2.1 Chemical reagents and cell lines

HPLC-grade methanol (MeOH) was purchased from Merck (Darmstadt, Germany). All the standards (UPLC-ESI-MS/MS) were purchased from Shanghai ZZBIO Co., Ltd. (Shanghai, China) and Extrasynthese (Lyon, France). Hydrochloric acid was bought from Xinyang Chemical Reagent (China). HPLC standards were purchased from Chengdu Biopurify Phytochemicals Ltd. (Chengdu, China; catalogue numbers can be found at: https://www.phytopurify.com/). Gallic acid, chlorogenic acid, quercetin, Folin-Ciocalteu reagent, 2,4,6-Tri(2-pyridyl)-s-triazine (TPTZ), formic acid, 2-thiobarbituric acid, and 2,2-diphenyl-1-picrylhydrazyl (DPPH) were purchased from Sigma-Aldrich (São Paulo, Brazil). Ascorbic acid, ethylenediaminetetraacetic (EDTA), and absolute ethanol were purchased from Vetec (Duque de Caxias, Brazil). The cell lines (A549, HepG2, HCT8, and EA.hy926) were obtained from the Rio de Janeiro Cell Bank (Rio de Janeiro, Brazil).

### 2.2 Samples and preparation of aqueous extracts

To obtain the crude extract (CE), the acquired sample of *C. sinensis* was crushed (particle size of 60 Tyler mesh), and solid-liquid extraction was carried out with an aqueous solution of citric acid (0.2 mol/L; pH 1.8) in an extraction vat, protected from light and under constant agitation, following the optimised conditions (1:62.3 m/v, 60 °C for 15 min) proposed de Moura et al. (2022). The extract was filtered (qualitative filter paper) and divided into two portions. The first portion (CE) was stored under refrigeration and protected from light for spectrophotometric analysis and the evaluation of the chemical stability of the bioactive compounds. The second portion (CLE; crude lyophilised extract) was frozen at - 80 °C and subjected to freeze-drying under vacuum at 1,200 μmL Hg (Terroni, model LD 1500A, São Paulo, Brazil) for biological and chemical analysis.

### 2.3 Crude extract analysis

#### 2.3.1 Phenolic composition by UV–Vis spectrophotometry

The content of total phenolic compounds was determined using the Prussian Blue colourimetric method, according to Margraf et al. (2016), and the result was expressed as mg of gallic acid equivalent per 100 g dry weight sample (mg GAE/100 g DW). The total flavonoid (TF) and total anthocyanin (TA) contents were quantified using the methodology described by Lees and Francis (1972). These results were expressed as mg of quercetin equivalents per 100 g dry weight sample (mg QE/100 g DW) and mg cyanidin-3-*O*-glucoside equivalents per 100 g dry weight sample (mg CGE/100 g DW), respectively. The condensed tannin and *ortho*-diphenol contents were estimated using the methods proposed by Santos et al. (2018). The results were expressed as mg of (+)-catechin equivalents per 100 g dry weight sample (mg CE/100 g DW) and mg of chlorogenic acid equivalents per 100 g dry weight sample (mg CAE/100 g DW), respectively.

#### 2.3.2 Chemical antioxidant activity

Antioxidant activity (AA) was measured using five different colorimetric tests. The ferric reducing antioxidant power (FRAP; mg of ascorbic acid equivalents per 100 g dry weight, mg AAE/100 g DW); free radical scavenging activity in relation to DPPH (mg AAE/100 g DW); and the Fe^2+^ chelating ability (mg of EDTA equivalents per 100 g DW, mg EDTAE/100 g DW) were all evaluated using the methods proposed by Santos et al. (2017). The total reducing capacity, which measures hydrophilic and lipophilic antioxidants, was assessed using the method proposed by Margraf et al. (2016). The results were expressed as mg of quercetin equivalents per 100 g (mg QE/100 g DW). Finally, the hydroxyl radical scavenging activity was assessed using the method described by Mu et al. (2012), and the result was expressed as mg of AAE per 100 g (mg AAE/100 DW).

#### 2.3.3 Studies of stability of anthocyanins of the crude extract

##### 2.3.3.1 Effect of pH on *in vitro* antioxidant activity

The study of the stability of anthocyanins at different pH levels was carried out according to the method proposed by Pedro et al. (2016a), with adaptations. The CE was diluted with ultrapure water (1:1 v/v) to obtain an absorbance close to 1.00 (ʎ_max_ = 525 nm) and placed in an extraction tank (25 °C), protected from light, and under constant agitation. Aliquots of NaOH (5 mol/L) were added to the diluted CE, and the pH was monitored using a pH meter (Micronal B-474). After reaching the basic pH, reverse titration was carried out with aliquots of hydrochloric acid (HCl; 1.0 mol/L). The absorbances were recorded (ʎ_max_ = 525 nm) at different pH levels (acidic, basic, and neutral) during the titration, and the effect of pH on the antioxidant activity was assessed using the capture of the DPPH free radical method proposed by Santos et al. (2017).

##### 2.3.3.2 Effect of temperature on the anthocyanins’ stability

To evaluate the effect of temperature (60, 80, and 100 °C) on the CE stability, the method proposed by Escher et al. (2020) was used, with minor modifications. To obtain absorbance close to 1.00 (ʎ_max_ = 525 nm), the CE was diluted (1:1 v/v) with citrate/citric acid buffer solution (pH 3.6), distributed in test tubes, sealed, and immersed in a thermostatic bath (60 and 80 °C) and a glycerin bath (100 °C), and protected from light. The spectral profiles at the different temperatures were recorded at 30-min intervals for 8 h. From the absorbances obtained at a wavelength of 525 nm, the colour retention percentage (%R), anthocyanin degradation rate constant (k), and half-life time (t_1/2_) were calculated using Equations (1)–(3), respectively.

(1) $$\%R=\frac{A_{t}}{A_{0}}x\;100$$

(2) $$\ln\frac{A_{t}}{A_{0}}=-k\;x\;t$$

(3) $$\ln_{1/2}=\frac{\ln\;2\;}{k}$$

where, A_t_ = absorbance at time t; A_0_ = initial absorbance; t = determined times (min).

##### 2.3.3.3 Effect of light on colour attributes

The effect of light on the anthocyanins' stability in the CE was assessed according to the method proposed by Escher et al. (2020). The CE, diluted (1:1 v/v) in citrate/citric acid buffer solution (pH 3.6), was distributed in properly sealed test tubes, exposed to direct light from six white, fluorescent lamps (20 W), and stored in a closed wooden chamber with an internal temperature of 32 ± 2 °C. As a control, tubes in the same box were kept away from light. The maximum absorbance and spectral profiles were obtained every 12 h for four days (96 h). Stability was assessed using the absorbance obtained at the maximum wavelength (ʎ = 525 nm), colour intensity (CI), and the proportion of yellow (YP), red (RP), and blue (BP) pigments, which were calculated according to Equations (4)–(7), respectively.

(4) $$CI={Abs}_{420\;nm}+{Abs}_{520\;nm}+{Abs}_{620\;nm}$$

(5) $$\%\;YP=\left(\frac{{Abs}_{420\;nm}}{CI}\right)x\;100$$

(6) $$\%\;RP=\left(\frac{{Abs}_{520\;nm}}{CI}\right)x\;100$$

(7) $$\%\;BP=\left(\frac{{Abs}_{620\;nm}}{CI}\right)x\;100$$

### 2.4 Crude lyophilised extract analysis

#### 2.4.1 Phenolic compounds by UV–vis spectrophotometry

The methods used to estimate the content of total phenolic content (mg GAE/g of CLE), total flavonoids (mg QE/g of CLE), and total monomeric anthocyanins (mg CGE/g of CLE) were carried out according to the methods mentioned in section 2.3.1.

#### 2.4.2 Phenolic composition of the crude lyophilised extract by UPLC-ESI-MS/MS analysis

The phenolic compounds in the CLE were identified by ultra-performance liquid chromatography – electrospray ionisation – tandem mass spectrometry (UPLC-ESI-MS/MS), according to Shi et al. (2021). An aliquot (50 mg) of the CLE was solubilised in 1 mL of aqueous methanol solution (70% v/v), filtered through a membrane filter (0.22 μm, Anpel), and then 2 μL was injected into a UPLC-ESI-MS/MS system (UPLC, ExionLC™AD; MS, Applied Biosystems 6500 Triple Quadrupole). The UPLC conditions were as follows: C_18_ column (1.7 μm, 2.1 mm × 100 mm, WatersACQUITY BEH); solvent system, water (0.1% formic acid): methanol (0.1% formic acid); gradient program, 95:5 (v/v) at 0 min, 50:50 (v/v) at 6 min, 5:95 (v/v) at 12 min, held for 2 min, 95:5 (v/v) at 14 min; held for 2 min; flow rate, 0.35 mL/min; temperature, 40 °C.

**Fig. 1:**
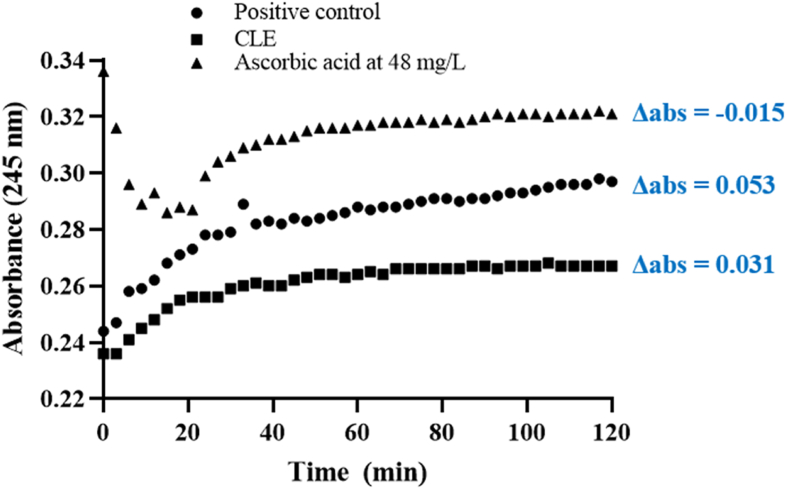
Effects of crude lyophilised extract (CLE) of *Camellia sinensis* var. *assamica* cv purple leaves. Zijuan on human plasma oxidation. (For interpretation of the references to colour in this figure legend, the reader is referred to the Web version of this article.)

Linear ion trap (LIT) and triple quadrupole (QQQ) scans were acquired using a triple quadrupole-linear ion trap mass spectrometer (QTRAP, QTRAP® 6500+ LC-MS/MS System) equipped with an ESI Turbo Ion-Spray interface, operating in positive ion mode, and controlled by Analyst 1.6.3 software (Sciex). The ESI source operation parameters were as follows: ion source, ESI+; source temperature 550 °C; ion spray voltage (IS) 5,500 V; and curtain gas (CUR) set at 35 psi. The anthocyanins were analysed using scheduled multiple reaction monitoring (MRM). The data acquisitions were performed using Analyst 1.6.3 software (Sciex). Multiquant 3.0.3 software (Sciex) was used to quantify all the metabolites. The mass spectrometer parameters, including the declustering potentials (DP) and collision energies (CE) for the individual MRM transitions, were performed with further DP and CE optimisation. A specific set of MRM transitions was monitored for each period according to the metabolites eluted within this period. The results were expressed in mg of each phenolic compound per g of crude lyophilised extract (mg/g of CLE). The Supplementary Materials (Table 1 and Fig. 1) contain the regression equation, upper and lower limits of quantification, the retention times, and a typical chromatogram of the extract using LC-MS and HPLC.

**Table 1:** Phenolic composition and antioxidant activity of crude extract of *Camellia sinensis* var. *assamica* cv. Zijuan.

Chemical composition and bioactivity	Content
*Chemical compounds (UV–Vis spectrophotometry)*	
Total phenolic content (mg GAE/100 g)	4,439 ± 69
Total flavonoids (mg QE/100 g)	285 ± 5
Total anthocyanins (mg CGE/100 g)	121 ± 1
*Ortho*-diphenols (mg CAE/100 g)	4,169 ± 175
Total flavonols (mg QE/100 g)	88 ± 4
*Antioxidant capacity*	
Capturing the DPPH free radical (mg AAE/100 g)	45,402 ± 1,019
Fe^2+^ chelating ability (mg EDTAE/100 g)	2,973 ± 173
Hydroxyl radical scavenging activity (mg AAE/100 g)	250,204 ± 2,635
Total reducing capacity (mg QE/100 g)	15,201 ± 334

#### 2.4.3 Analysis of phenolic compounds and caffeine

The flavan-3-ols, gallic acid, and caffeine were analysed by HPLC (Model Shimadzu LC-2010A; Shimadzu Corporation, Kyoto, Japan) following the methodology proposed by Xu et al. (2018). CLE (200 mg) was dissolved in 2 mL of methanol:water (70% v/v) and filtered on a 0.22 μm Millipore filter (Shanghai, China). The HPLC injection volume was 10 μL. A Diamonsil™ C_18_ column (250 × 4.6 mm I.D., 5 μm) was used at a temperature of 30 °C with an injection flow rate of 1 mL/min; the mobile phase was a mixture of A (2% acetic acid solution), and B (100% acetonitrile). The elution gradient was initially 6.5 % of mobile phase B, which was linearly increased to 15% in 16 min and 25% in 25 min; it was then reduced to 6.5% in 30 min and maintained until 35 min of injection. A Shimadzu SPD ultraviolet detector (Shimadzu Corporation) was used to detect compounds at 280 nm. The results were expressed as mg per g of CLE (mg/g of CLE).

#### 2.4.4 Antioxidant activity

The CLE was evaluated against the following antioxidant methods: FRAP (mg AAE/g of CLE); free radical scavenging activity in relation to DPPH (mg AAE/g of CLE); Fe^2+^ chelating ability (mg EDTAE/g of CLE); and the inhibition of lipid peroxidation induced by the Fe^2+^ in fowl egg yolk in a buffered system (pH 7.4) with different concentrations of CLE (400–2,000 mg/L). The results were expressed as the required concentration of CLE for 50% inhibition of lipid peroxidation (IC_50_) (Margraf et al., 2016).

#### 2.4.5 Effect of CLE on human cell viability and cellular antioxidant activity

The cell viability was assessed by MTT assay after 48 h of CLE treatment (15.0; 38.0; 76.0; 152.0; 228.0, and 380.0 μg GAE/mL) in human lung adenocarcinoma epithelial (A549); ileocecal colorectal adenocarcinoma (HCT8); hepatocellular carcinoma (HepG2); and somatic cell hybrid (EA.hy926) cells. Briefly, the cells were seeded in 96-well plates at a density of 1 × 10^4^ cells per well. Twenty-four hours later, they were treated for 48 h with the CLE at 0–380 μg GAE/mL. At the end of the 48 h of treatment, 0.5 mg/mL MTT was added and incubated at 37 °C for 4 h. DMSO was added to dissolve the crystalline formazan product, and the absorbance at 570 nm was measured. The IC_50_, GI_50_, and LC_50_ parameters were performed in accordance with the method described by do Carmo et al. (2018).

The cellular antioxidant activity assay used DCFH-DA (2ʹ,7ʹ-dichlorofluorescein diacetate) to detect oxidative products from the cell lines. The A549, HCT8, HepG2, and EA.hy926 cells were seeded at 6 × 10^4^/well and treated for 1 h at 37 °C with the CLE diluted in a DCFH-DA solution (25 mmol/L) at 7.5, 15.0, and 38.0 μg GAE/mL. The cells were treated with 15 μmol/L of H_2_O_2_ for the positive control. The cells were only treated with the culture medium for the negative control. After incubation, the ROS levels were measured by fluorometric detection using a spectrophotometer (excitation 485 nm; emission 538 nm). The DCF fluorescence intensity was proportional to the intracellular ROS generation (Granato et al., 2022).

#### 2.4.6 Plasma protection against oxidation

Human plasma was obtained from a healthy volunteer (male, 39 years old, body mass index <25 kg/m^2^, O^+^ blood type) after approval by the Research Ethics Committee (protocol 2023_02_01_S&E) and obtaining written consent from the blood donor. The assay followed the experimental setup and conditions established by Corrigan et al. (2023). For this purpose, the CLE was diluted 100-fold in phosphate saline buffer (5 mmol/L, NaCl at 150 mmol/L, pH 7.35). The antioxidant activity was compared to a standard curve of ascorbic acid (12–120 mg/L, R^2^ = 0.9923) by measuring the formation of conjugated dienes at 245 nm for 120 min at 3 min intervals. The difference in absorbance (Δabs) between time 120 min and 0 min was calculated, and a graphical illustration was created (X-axis: time [min], and Y-axis: absorbance at 245 nm). The experiments were repeated five times.

### 2.5 Statistical analysis

All the analyses were carried out in triplicate, with the results expressed as mean and standard deviation. The Brown-Forsythe test was employed to access homoscedasticity, and one-way variance analysis (ANOVA) was used to investigate the difference between the means, followed by Fisher's test of least significant difference (*p* ≤ 0.05). TIBCO Statistica v. 13.3 (TIBCO Software Ltd, Palo Alto, CA, USA) software was used.

## 3 Results and discussion

### 3.1 Determination of polyphenols and chemical antioxidant activity of the crude extract

The results of the spectrometric analysis of the chemical composition and antioxidant activity of the CE are shown in Table 1. The CE showed a diverse chemical composition, with the *ortho*-diphenol content standing out (4,169 ± 175 CAE/100 g), followed by the total flavonoid content (285 ± 5 mg QE/100 g), total anthocyanins (121 ± 1 mg CGE/100 g), and total flavonols (88 ± 4 mg QE/100 g). The total phenolic content in the CE was 4,439 ± 69 mg GAE/100 g, which was lower than the value reported in a study by Gao et al. (2018) that evaluated the phenolic content of the aqueous extract of Zijuan tea (42,259 ± 12.09 mg GAE/g). Studies of purple tea derived from *C. sinensis* are still scarce, and due to the diversity of the methods of presenting the results, it is not easy to compare them. For example, Kilel et al. (2013) obtained their results as a percentage of total polyphenols, with the highest average value of 25.9% among the analysed samples. Kerio et al. (2013) also evaluated their purple tea samples as a percentage of total phenolic content, and they obtained an average of 22% polyphenols in purple leaf-coloured samples. Despite the different ways the results were presented, extracts obtained from the purple leaves of *C. sinensis* are an essential source for extracting phenolic compounds.

Table 1 demonstrates that the CE showed antioxidant potential in all the tested methods. The presence of different phenolic compounds gave the extract antioxidant properties, either through the donation of the proton or the electrons or the ability to chelate transition metals, such as iron. In a study carried out by de Moura et al. (2022), the acidified ethanolic extract showed levels of 50,957 ± 835 mg AAE/100 g of Zijuan tea for the evaluation of capturing the DPPH free radical and 16,995 ± 356 for total reducing capacity. These values are slightly higher than in the present study, which were 45,402 ± 1,019 mg AAE/100 g for capturing the DPPH free radical and 15,201 ± 334 mg QE/100 g for total reducing capacity. This behaviour can be explained by the increased polarity of the extracting solvent and, consequently, a higher content of bioactive compounds extracted (5,016 ± 66 mg GAE/100 g; total phenolic content), while in the present study, the total phenolic compound content was 4,439 ± 69 mg GAE/100 g.

Compared to other teas derived from *C. sinensis*, the AE showed superior antioxidant indices, as in the study carried out by Armstrong et al. (2020), who obtained the following average AA levels for the aqueous extract of Pu-erh tea: 2,493 ± 69 mg AAE/100 g and 1,771 ± 77 mg AAE/100 g for the DPPH free radical capture and FRAP analyses, respectively. In the same study, the authors reported the antioxidant activity of mate (2,882 ± 1,630 mg AAE/100 g), black (2,810 ± 94 mg AAE/100 g), and green tea (7,613 ± 110 mg AAE/100 g) for capturing the DPPH free radical. These values were lower than those observed for purple tea in the present study, demonstrating the extract's high antioxidant potential in terms of the DPPH radical capture method.

### 3.2 Phenolic composition and *in vitro* bioactivity of CLE

The phenolic composition and antioxidant activity of the CLE were evaluated by chemical, biological and chromatographic tests, and the results are shown in Table 2. It is possible to observe that the lyophilization process partially degraded polyphenols compared to the crude extract: for example, the total phenolic content decreased by 14%, and the total flavonoid and anthocyanin contents decreased by 47% and 58%, respectively. Although lyophilization is a recognized method of preservation, we showed that it negatively affects the content of polyphenols in purple tea extract, and this is in line with previous observations (Cheaib et al., 018).

**Table 2:** Chemical composition and antioxidant activity of crude lyophilised extract of *Camellia sinensis* var. *assamica* cv. Zijuan.

Chemical composition and bioactivity	Content
*Chemical compounds (UV–Vis spectrophotometry)*	
Total phenolic content (mg GAE/g)	38 ± 2
Total flavonoids (mg QE/g)	1.5 ± 0.01
Total anthocyanins (mg CGE/g)	0.5 ± 0.01
*Ortho*-diphenols (mg CAE/g)	15 ± 1.5
*Antioxidant capacity*	
Free radical scavenging activity - DPPH (mg AAE/g)	110 ± 4
FRAP (mg AAE/g)	76 ± 1
Fe^2+^ chelating ability (mg EDTAE/g)	8 ± 0.5
Inhibition of lipoperoxidation (IC_50;_ mg/L)	1,731 ± 15
Protection against induced human plasma oxidation (mg AAE/g)	635 ± 30
*Chemical composition by HPLC and UPLC-ESI-MS/MS (μg/g)*	
(−)-Epigallocatechin gallate	15,323 ± 1
(−)-Epicatechin gallate	8,031 ± 25
Caffeine	6,532 ± 1
(−)-Epigallocatechin	4,978 ± 18
Gallic acid	1,709 ± 36
(−)-Gallocatechin	561 ± 24
(−)-Catechin gallate	542 ± 55
(−)-Epicatechin	520 ± 6
(+)-Catechin	398 ± 60
Procyanidin B2	324 ± 15
Rutin	323 ± 37
Quercetin-3-*O-*glucoside	244 ± 10
(−)-Gallocatechin gallate	177 ± 23
Procyanidin B3	146 ± 13
Delphinidin-3-*O*-galactoside	107 ± 6
Procyanidin C1	77 ± 4
Kaempferol-3-*O*-rutinoside	51 ± 9
Procyanidin B1	40 ± 0.1
Pelargonidin-3-*O*-galactoside	27 ± 5
Delphinidin-3-*O*-(6-*O*-*p*-coumaroyl)-glucoside	25 ± 3
Delphinidin-3-*O*-5-*O*-(6-*O*-coumaroyl)-diglucoside	10 ± 0.5
Delphinidin-3,5-*O*-diglucoside	8 ± 0.5
Peonidin-3-*O*-(6-*O*-*p*-coumaroyl)-glucoside	7 ± 1
Dihydromyricetin	6.5 ± 0.3
Peonidin-3-*O*-galactoside	3.3 ± 0.5
Naringenin-7-*O*-glucoside	2.2 ± 0.1
Petunidin-3-*O*-arabinoside	1.8 ± 0.3
Cyanidin-3-(6-*O*-*p*-caffeoyl)-glucoside	1.8 ± 0.3
Cyanidin-3-*O*-glucoside	1.6 ± 0.1
Cyanidin-3,5*-O-*diglucoside	1.1 ± 0.1
Procyanidin A2	0.84 ± 0.22
Kaempferol-3-*O*-rhamnoside	0.75 ± 0.01
Narigenin	0.42 ± 0.02
*Total identified (μg/g)*	*40,180*

Note: IC_50_ minimum concentration necessary to inhibit 50% of lipoperoxidation.

Two chromatographic methods were used to increase the range of identified chemical compounds; a total of 33 compounds were quantified. At higher concentrations, procyanidin B2 (324 ± 15 μg/g), rutin (323 ± 37 μg/g), quercetin-3-*O*-glucoside (244 ± 10 μg/g) and delphinidin-3-*O*-galactoside (107 ± 6 μg/g) were notable in the results obtained by UPLC-ESI-MS/MS. When evaluating the main catechins present in the CLE by HPLC, (−)-epigallocatechin gallate (15,323 ± 1 μg/g), (−)-epigallocatechin gallate (8,031 ± 25 μg/g) and (−)-epigallocatechin (4,978 ± 18 μg/g) were the primary flavanols, confirming (−)-epigallocatechin gallate as the predominant polyphenol in unfermented *C. sinensis* tea, and corroborating the results obtained by Granato et al. (2014), Xu et al. (2018) and Zhang et al. (2019).

The purple leaves of *C. sinensis* give the tea a complex mixture of phenolic compounds, as shown in the present study. The anthocyanins that provide the leaves with this colour were identified, such as delphinidin-3-*O*-galactoside (107 ± 6 μg/g), cyanidin-3-(6-*O*-*p*-caffeoyl)-glucoside (1.8 ± 0.3 μg/g) and pelargonidin-3-*O*-galactoside (27 ± 5 μg/g). Lv et al. (2015), identified anthocyanins in Zijuan tea infusions. They detected the highest concentration of cyanidin-3-*O*-galactoside, as well as anthocyanidins such as delphinidin (4.31 μg/mL), cyanidin (18.5 μg/mL), pelargonidin (14.9 μg/mL), peonidin (23.8 μg/mL) and malvidin (11.1 μg/mL). Shi et al. (2021) identified thirty-three anthocyanins in their study, with delphinidin 3-*O*-galactoside and cyanidin 3-*O*-galactoside being the most abundant. The aforementioned authors highlighted the fact that the anthocyanidins cyanidin, delphinidin, malvidin, pelargonidin, peonidin, and petunidin were found in the 3-*O* glycosidically-bound forms. They played a fundamental role in the purple colouration of the tea leaves and being structurally stable compared to anthocyanidins.

The antioxidant activity of the CLE was evaluated using different chemical and biological assays, and the results are shown in Table 2. CLE inhibited induced lipid oxidation in a dose-dependent behaviour: 19 ± 2% (400 mg/L), 24 ± 3% (800 mg/L), 46 ± 1% (1,600 mg/L) and 58 ± 3% (2,000 mg/L), as well as IC_50_ of 1,731 ± 15 mg/L, showing that CLE was effective in donating hydrogen atoms to free radicals produced by Fenton reactions in the medium. Furthermore, CLE scavenged DPPH radicals, reduced iron ions (FRAP), and chelated Fe^2+^, highlighting its single-electron transfer and metal-chelating abilities.

When human plasma was challenged with CuCl_2_ to induce the lipid peroxidation of polyunsaturated fatty acids (Fig. 1), CLE showed protective effects (635 ± 30 mg AAE/g). Compared to the positive control (maximum production of conjugated dienes), it is possible to observe that 100-fold diluted CLE had lower Δabs (0.031) compared to the positive control (Δabs = 0.053) but higher than ascorbic acid at 48 mg/L (Δabs = −0.015). These values corroborate the results of chemical antioxidant assays (i.e., inhibition of egg yolk lipid peroxidation), highlighting the effectiveness of CLE in donating hydrogen atoms to free radicals. According to Corrigan et al. (2023), the ability of phenolic compounds to eliminate free radicals and reduce pro-oxidant metals may explain the protection of human plasma from oxidation and highlight the antioxidant potential of phenolic compounds in CLE. In their study, Corrigan et al. (2023) observed that chamomile tea (227 mg AAE/L) and chamomile tea beverage supplemented with *t*-resveratrol and quercetin (379 mg AAE/L) protected human plasma against induced oxidation *in vitro*. These results show that plant extracts, such as CLE, are a viable alternative for the industry to replace synthetic antioxidants and colourants; however, bioavailability studies covering digestion and absorption processes and *in vivo* studies are needed to evaluate the functionality of extracts.

#### 3.2.1 Cell viability and intracellular antioxidant activity

**Fig. 2:**
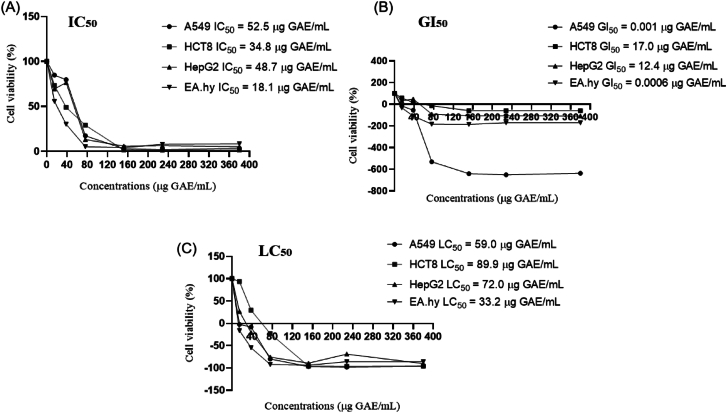
Cell viability and dose-response effect after 48 h exposure to crude lyophilised extract (CLE) of *Camellia sinensis* var. *assamica* cv purple leaves. Zijuan in A549, HCT8, HepG2, and Ea.hy926 cell lines. (A) IC_50_: concentration of the agent that inhibits cell growth by 50%; (B) GI_50_: concentration of the agent that inhibits growth by 50%, relative to untreated cells; (C) LC_50_: concentration of the agent that results in a net loss of 50% cells, relative to the number at the start of treatment.

The MTT cell proliferation assay tests the rate of cell proliferation and, conversely, cell viability reduction when metabolic events lead to apoptosis or necrosis (Suganya et al., 2022). The treatment with the lowest concentration of CLE revealed a decrease in cell viability (IC_50_ from 18.1 to 52.5 μg GAE/mL), proliferation (GI_50_ from 0.0006 to 17.0 μg GAE/mL) and cytotoxicity (LC_50_ from 33.2 to 89.9 μg GAE/mL) in a dose-dependent manner in both cancer and normal cells (Fig. 2). The CLE presented a large variety of flavonoids, e.g. (−)-epicatechin gallate, (−)-epigallocatechin gallate, and (−)-epigallocatechin. These phenolic compounds can exert cytotoxic effects by suppressing several phase-I metabolising enzymes, such as cytochrome P450, which metabolically activates numerous procarcinogens and causes carcinogenesis and the inhibition of enzymes like xanthine oxidase, COX (cyclooxygenase), and LOX (lipoxygenase), which are associated with inflammatory and cancerous diseases (Rajasekar et al., 2023). Interestingly, the EAhy926 non-cancer cell seemed to be most sensitive to CLE (Fig. 2) because it presented the lowest IC_50_ values (18.1 μg GAE/mL), GI_50_ (0.0006 μg GAE/mL), and LC_50_ (33.2 μg GAE/mL), compared with the cancer cells. Despite this higher sensitivity on non-cancer cells, additional analyses are required to guarantee the product's toxicological safety when developing new food ingredients. These steps include, for example, the understanding of other CLE/CE properties, such as solubility and stability; technologies involved in manufacturing the food, *in vitro* experiments with digested extracts, animal experiments, and the validation of the efficacy of functional foods in human intervention studies with standard scientific protocols (Azevedo et al., 2023). Indeed, phenolic compounds may be degraded during gastrointestinal digestion due to the intestinal environment's pH, digestive enzymes, biliary acids, and food matrix features, which can modify the chemical structures, resulting in new compounds with different bioavailability and biological activities (Tomas et al., 2024). In other words, the digestive process and interaction with gut microbiota can change the bioavailability of compounds and significantly affect the bioactivity and cytotoxicity profile of bioactive-rich extracts.

**Fig. 3:**
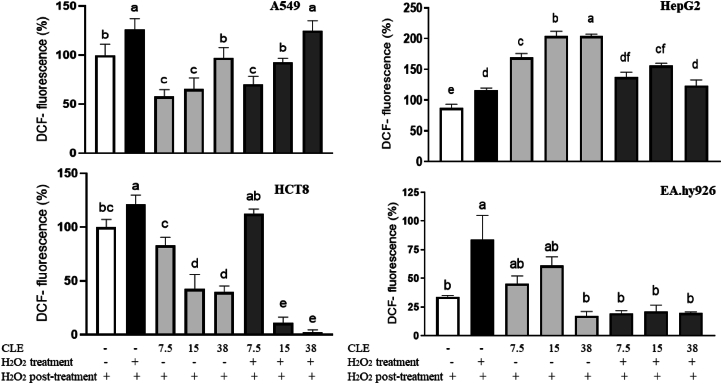
Effects of the crude lyophilised extract (CLE; 7.5–38 μg GAE/mL) of purple leaves on the intracellular ROS generation in A549, HepG2, HCT8, and Ea.hy926 cells. Quantitative data are the mean ± standard deviation (n = 4). Different letters represent statistically significant differences (*p* ≤ 0.05).

Optimal intracellular ROS concentrations provide a permissive oxidative environment for cellular proliferation (do Carmo et al., 2021). For the EA.hy926 normal cells, and the HCT8 (in 15.0 and 38.0 μg GAE/mL) and A549 (in 7.5 and 15.0 μg GAE/mL) cancer cells, the CLE exerted antioxidant intracellular activity by reducing the oxidative stress induced by H_2_O_2_ (Fig. 3). Li et al. (2021) observed that procyanidin B2 and rutin, phenolic compounds found in the CLE in the present study (323.6 ± 15.1 μg/g and 322.6 ± 36.9 μg/g, respectively)*,* increased the expression of nuclear factor erythroid 2–related factor 2 (Nrf2), which is the primary regulator of the antioxidant response that protects cells from ROS-induced damage, and activated Erk1/2 signalling, which promotes Nrf2-mediated protection. H_2_O_2_ levels below this range can lead to the disruption of intracellular signalling, which can result in the loss of homeostasis, with a consequent reduction in cell viability. In contrast, phenolic compounds can act as pro-oxidant agents, and too high levels of H_2_O_2_ can also reduce cell viability by stimulating apoptosis pathways that can lead to cell death (do Carmo et al., 2021). In the present study, the CLE induced ROS generation in HepG2 cancer cells and did not protect them against the oxidative stress induced by H_2_O_2_. Therefore, the antioxidant and pro-oxidant behaviour of the CLE may explain the cytotoxic effects observed in the cell viability assay, and we would hypothesise that this property can be attributed to the phenolic compound, especially the flavonoids in the CLE.

### 3.3 Studies of stability of anthocyanins of the anthocyanin extract

#### 3.3.1 Effect of pH and *in vitro* antioxidant activity

**Fig. 4:**
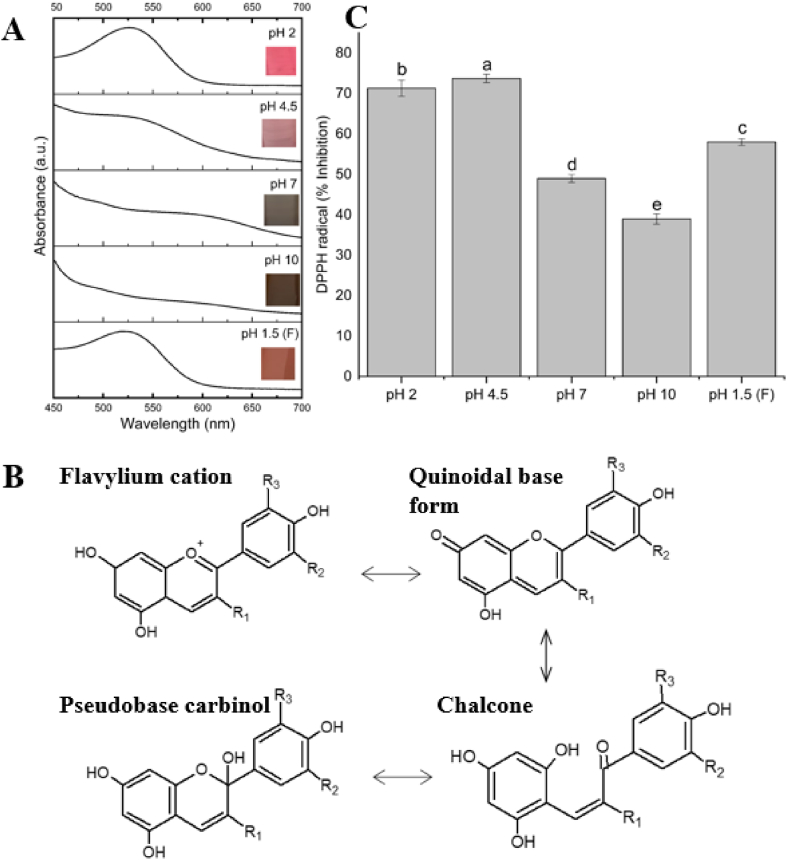
Effect of pH on the stability and reversibility of anthocyanins present in the crude extract of *Camellia sinensis* var. *assamica* cv. Zijuan. (A) Absorption spectrum of extracts between pH 2 and 10, and reversibility at pH 1.5 (F: final) with colour variations of the extracts. (B) Molecular structures found in the aqueous solution at different pH values. (C) Effect of pH on antioxidant activity of anthocyanins present in the extract. Different letters represent statistically significant differences (*p* ≤ 0.05). (For interpretation of the references to colour in this figure legend, the reader is referred to the Web version of this article.)

The effect of varying the pH on the *in vitro* antioxidant activity of the CE is shown in Fig. 4. The spectral profile of the CE at pH 2, 4.5, 7, 10, and 1.5 (final pH; obtained by reserve titration) is shown in Fig. 4A, together with the colours obtained from the extract at each pH, which are pink (pH 2), light pink (pH 4.5), grey (pH 7), brown (pH 10) and pink again (pH 1.5 F). It is possible to observe the progressive decrease in absorbance obtained at a wavelength of 525 nm and the loss of the characteristic peak at pH 7 and 10. Anthocyanins (Fig. 4B) exist as flavylium cations (AH^+^) in an acidic environment and are red. As the pH increases, they deprotonate and form a neutral quinoidal form, which is an unstable compound susceptible to nucleophilic attack by water molecules, resulting in the development of the pseudobases carbinol and chalcone; this process is reversible with a change in pH (Pedro et al., 2016; Escher et al., 2020). The chemical degradation of anthocyanins causes irreversible loss when the heterocyclic ring ruptures. When performing reverse titration, it was found that the anthocyanins present in the extract showed reversibility in the displacement of the equilibrium structure, with a recovery of 80% of the initial absorption.

The AA evaluated by the DPPH free radical capture method is shown in Fig. 4C and is expressed as % inhibition. All the responses demonstrated inhibition against the DPPH radical and showed a significant difference (p ≤ 0.05). The CE showed the highest percentage of inhibition at pH 4.5 (73% inhibition), followed by the CE at pH 2.0 (71% inhibition), and pH 1.5 (F) with 58% inhibition. The lowest percentages of inhibition were observed at pH 10 (39% inhibition) and pH 7 (49% inhibition). With changes in pH, the deprotonation of the chemical structure may influence an extract's ability to capture the free radicals present in the reaction, corroborating the results obtained in this study. Escher et al. (2020) assessed the stability of anthocyanins in the aqueous extract of *blue petals of C. ternatea (butterfly pea)*. They obtained results similar to those of the present study, with a slight increase in AA at pH levels close to 4 (53–59% between pHs 2.25 and 5.25) and a decrease to 43% at pH 10.

#### 3.3.2 Effect of temperature

**Fig. 5:**
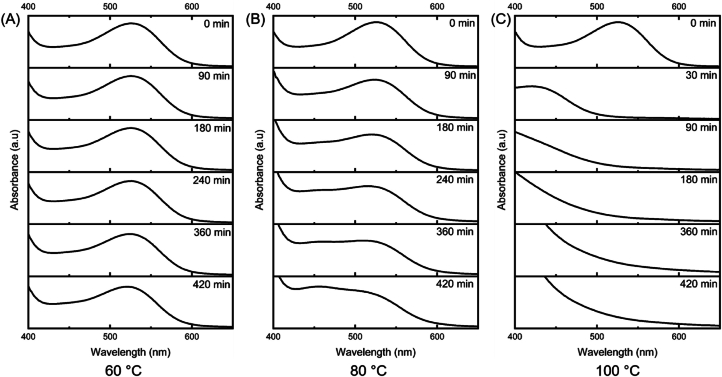
Effect of temperature on the stability of anthocyanins present in crude *Camellia sinensis* var. *assamica* cv extract. Zijuan for 420 min. Absorption spectrum of extracts at 60 °C (A), 80 °C (B), and 100 °C (C).

The anthocyanins' stability in the CE was assessed at 60, 80, and 100 °C for 420 min. The kinetic parameters (percentage of colour retention, rate constant of anthocyanin degradation, and half-life time) are shown in Table 3. The absorbance was recorded at 525 nm, and the spectral profile (400–650 nm) can be seen in Fig. 5. When evaluating the data shown in Table 3, the temperature of 60 °C (k = 1. 9 × 10^−4^) degraded only 8% of the anthocyanins present in the CE, demonstrating high stability at this temperature. A time of 3,600 min (60 h) was required to degrade 50% of the anthocyanins, while when exposed to 80 °C (k = 5.8 × 10^−4^), a loss of 20% was observed, with a half-life of 1,260 min (21 h). The temperature of 100 °C (k = 3.3 × 10^−3^) was highly detrimental to the stability of the anthocyanins, as only 7% of the colour retention was preserved, and the half-life time was 8 min.

**Table 3:** Kinetic parameters of the effect of temperature on the stability of the anthocyanins in crude extract of *Camellia sinensis* var. *assamica* cv. Zijuan.

Kinetic parameters	Temperature		
	60 °C	80 °C	100 °C
Colour retention (%)	92	80	7
k	1.9 × 10^−4^	5.8 × 10^−4^	3.3 × 10^−3^
t_1/2 (min)_	3600	1260	8

k: rate constant of anthocyanin degradation; t_1/2_ half-life time.

Studies in the literature show that natural extracts rich in anthocyanins show instability and degradation of compounds at temperatures above 60 °C. Migliorini et al. (2019) exposed the anthocyanin extract of red chicory ((*Cichorium intybus* L.) to 60 °C. They obtained a half-life time of 74.23 h, while temperatures of 80 and 100 °C decreased the half-life time to 9.55 h and 2.25 h, respectively. Pedro et al. (2016) assessed the stability of anthocyanins derived from purple basil (*Ocimum basilicum* L.). They obtained half-lives of 53.82 h, 27.56 h, and 12.62 h when exposing the extracts to 60, 80 and 90 °C, respectively. The CE showed similar behaviour to the chicory and purple basil extracts in the aforementioned studies when the extract was exposed to a temperature of 60 °C but was more sensitive to degradation at higher temperatures (close to 100 °C).

The spectral profiles of the extract at different temperatures (Fig. 5) corroborate the kinetic data obtained. The decrease in absorbance at a wavelength of 525 nm can be observed at all temperatures, with greatest intensity at temperatures of 80 °C (Figs. 5B) and 100 °C (Fig. 5C). Results relating to the stability of anthocyanins are fundamental for preserving the structure and desired bioactivity of compounds, to apply extracts in food matrices or in the pharmaceutical industry, which may undergo thermal processing for preparation and/or preservation.

#### 3.3.3 Effect of light

**Fig. 6:**
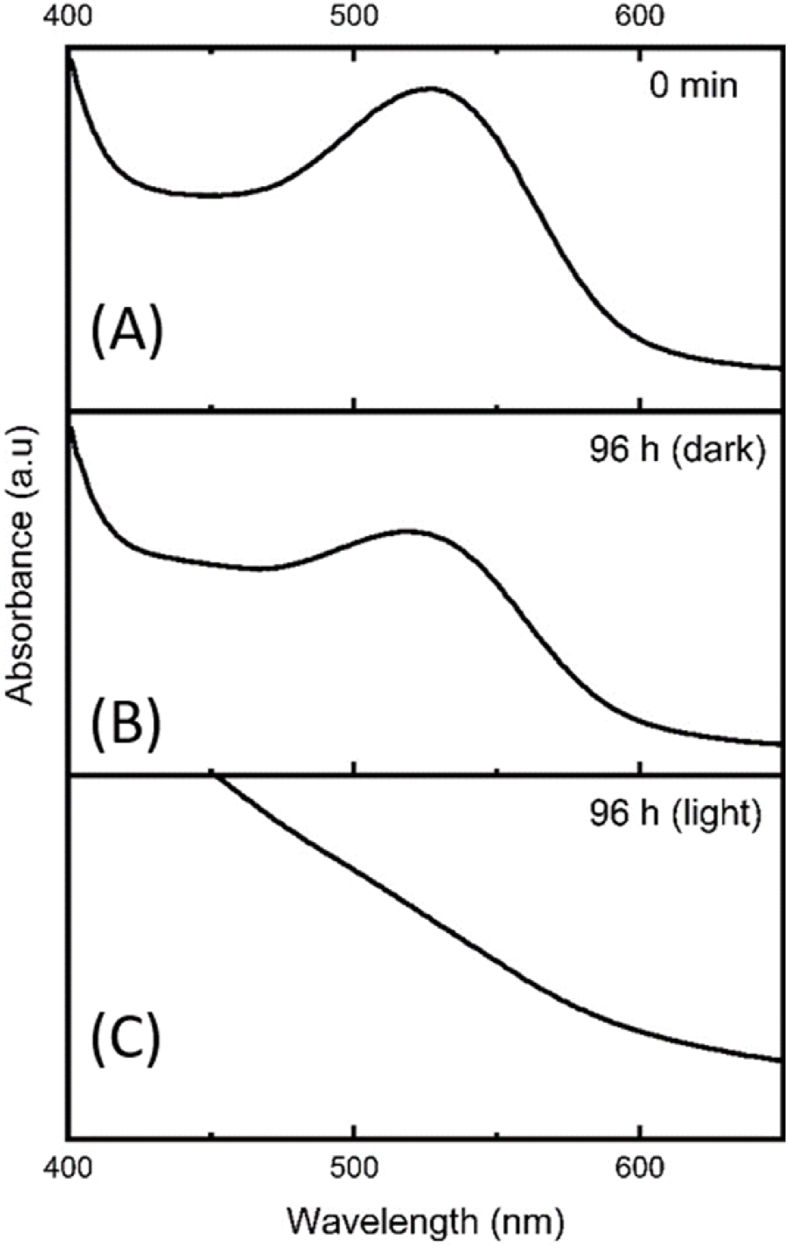
Effect of light on the stability of anthocyanins in the crude *Camellia sinensis* var. *assamica* cv. Zijuan extract for 96 h. (A) Absorption spectrum of extracts at time 0 h (start of experiment). (B) Absorption spectrum after 96 h without exposure to light (dark). (C) Absorption spectrum of extracts after 96 h of exposure to light.

The effect of light as a function of time on the stability of the anthocyanins present in the CE was evaluated over 96 h. The percentage of yellow, red, and blue pigments was used to assess the degradation of anthocyanins, and the results are shown in Table 4. The spectral profile (400–700 nm) for the initial and final extract (96 h; dark and light) is shown in Fig. 6. The results showed a significant difference (p ≤ 0.05). The percentage of red pigments varied from 53 to 31%, while the percentage of yellow pigments rose from 38 to 57% after 96 h of exposure to light. After 12 h of exposure, there was an increase in the proportion of red pigments; in contrast, yellow pigments were not altered, confirming that 12 h of exposure to light was not capable of degrading anthocyanins.

**Table 4:** The proportion of red, yellow and blue pigments in a crude extract of *Camellia sinensis* var. *assamica* cv. Zijuan exposed to light and protected from light over a period of 96 h.

Time	Red pigments (%*)		Yellow pigments (%*)		Blue pigments (%*)	
	Light	Dark	Light	Dark	Light	Dark
0 min	53 ± 0.1^b^	53 ± 0.1c	38 ± 0.4^h^	38 ± 0.4^h^	9 ± 0.3^cd^	9 ± 0.3^a^
12 h	56 ± 0.3^a^	57 ± 0.1^a^	38 ± 0.2^h^	37 ± 0.1^g^	6 ± 0.1^e^	6 ± 0.1^c^
24 h	51 ± 0.5^c^	54 ± 0.1^b^	41 ± 0.5^g^	38 ± 0.1^g^	8 ± 0.3^d^	7 ± 0.5^b^
36 h	46 ± 0.4^d^	54 ± 0.6^b^	45 ± 0.4^f^	39 ± 0.1^f^	9 ± 0.6^cd^	7 ± 0.5^bc^
48 h	43 ± 0.9^e^	53 ± 0.6^c^	47 ± 0.9^f^	40 ± 0.1^e^	10 ± 0.5^bc^	7 ± 0.7^b^
60 h	39 ± 0.7^f^	51 ± 0.8^d^	50 ± 0.7^d^	41 ± 0.2^d^	11 ± 0.7^b^	8 ± 0.7^ab^
72 h	38 ± 0.7^g^	50 ± 0.6^d^	52 ± 0.8^c^	42 ± 0.2^c^	11 ± 0.9^b^	8 ± 0.7^ab^
84 h	34 ± 0.8^h^	50 ± 0.7^d^	55 ± 1.5^b^	43 ± 0.3^b^	11 ± 0.9^ab^	7 ± 0.6^bc^
96 h	31 ± 0.4^i^	47 ± 0.7^e^	57 ± 1.2^a^	45 ± 0.2^a^	12 ± 1.1^a^	8 ± 0.8^b^
*p*-Value^1^	0.951	0.964	0.762	0.727	0.871	0.915
*p*-Value^2^	<0.001	<0.001	<0.001	<0.001	<0.001	0.007

Note: *Mean ± standard deviation (n = 3). ^1^Probability values obtained with the Brown-Forsythe test for homogeneity of variances; ^2^Probability values obtained by one-way ANOVA; Different letters in the same column represent statistically different results (*p* ≤ 0.05) according to Fisher's test.

Samples in the same box without exposure to light were used as a control to assess only the influence of light on the degradation of anthocyanins. It was possible to observe a slight change in the proportion of red pigments (53–47%) and yellow pigments (38–45%). The proportion of blue pigments varied from 9 to 12% when exposed to light for 96 h. When evaluating the spectral profile of the samples (Fig. 6), the characteristic peak at the maximum wavelength referring to anthocyanins can be seen in the spectral profile of the initial extract (Fig. 6A). The extract stored in the dark (Fig. 6B). For the extract exposed to light, this characteristic peak was lost (Fig. 6C), confirming the degradation of anthocyanins.

These results show that the prolonged exposure of the CE to light caused the photodegradation of the anthocyanins present in the medium, generating yellow compounds, such as *cis*-chalcones, through the rupture of the heterocyclic ring and making the reaction irreversible for some anthocyanins. Escher et al. (2020) assessed the stability of anthocyanins in the aqueous extract of *C. ternatea* blue petals (pH 3.6). They noted that 56% of the anthocyanins were degraded when assessing the percentage of colour retention over 232 h. Contreras-Lopez et al. (2014) studied the impact of light on the stability of anthocyanins present in ethanolic blackberry extracts (*Rubus fructicosus*). They showed that exposure to light accelerates the destruction of anthocyanin pigments, with a 76% decrease in the concentration of monomeric anthocyanins.

Thus, these results reinforce the need for care when handling foods enriched with anthocyanin extracts, as well as suitable packaging that can block light from the visible spectrum, and especially from the ultraviolet field of the spectrum, to store the products and guarantee the quality and maintenance of the chemical structure of the anthocyanins (Enaru et al., 2021).

## 4 Conclusions

A lyophilised crude extract of *C. sinensis* cv. Zijuan, obtained using a simple, low-cost, and non-toxic method, showed quantified levels of different classes of phenolic compounds, *in vitro* antioxidant activity, reversible equilibrium of anthocyanins, and antioxidant activity with pH variation. Photodegradation of the anthocyanins was observed after 96 h of exposure to light, and thermal degradation was observed when the extract was exposed to temperatures above 80 °C. The lyophilised crude extract showed a diverse phenolic composition and antioxidant properties, such as inhibition of lipid peroxidation and human plasma protection against copper-induced oxidation. The *in vitro* cell analyses revealed that the antioxidant and pro-oxidant dual face behaviour of the CLE interfered with cell viability, exerting cytotoxic effects. The results indicate a technological potential of CLE to be applied in food and pharmaceutical products, possibly as a synthetic colourant and antioxidant. However, storage and handling conditions must be carefully checked to prevent prolonged exposure to light and high temperatures from degrading anthocyanins. It is suggested that further *in vivo* bioavailability and bioaccessibility studies are performed.

## CRediT authorship contribution statement

**Cristiane de Moura:** Conceptualization, Methodology, Data curation, Formal analysis, Writing – review & editing, Writing – original draft. **Mariana Araújo Vieira do Carmo:** Methodology, Formal analysis, Writing – original draft. **Yong-Quan Xu:** Methodology, Investigation, Resources, Formal analysis, Writing – original draft. **Luciana Azevedo:** Methodology, Formal analysis, Data curation, Resources, Writing – review & editing. **Daniel Granato:** Conceptualization, Methodology, Software, Validation, Investigation, Formal analysis, Resources, Data curation, Writing – review & editing, Supervision, Project administration, Funding acquisition.

## Declaration of competing interest

The authors declare that they have no known competing financial interests or personal relationships that could have appeared to influence the work reported in this paper.

## Data Availability

Data will be made available on request.

## References

[bib1] Alappat B., Alappat J. (2020). Anthocyanin pigments: beyond aesthetics. Molecules.

[bib2] Armstrong L., Araújo Vieira do Carmo M., Wu Y., Antônio Esmerino L., Azevedo L., Zhang L., Granato D. (2020). Optimizing the extraction of bioactive compounds from pu-erh tea (Camellia sinensis var. assamica) and evaluation of antioxidant, cytotoxic, antimicrobial, antihemolytic, and inhibition of α-amylase and α-glucosidase activities. Food Res. Int..

[bib43] Azevedo L., Granato D., Maltarollo V.G., Gonçalves J.E. (2023). A mosaic-structured framework applied in the healthy food design: insights from integrated in silico and in vitro approaches. Curr. Opin. Food Sci..

[bib3] Cheaib D., El Darra N., Rajha H.N., El-Ghazzawi I., Maroun R.G., Louka N. (2018). Effect of the extraction process on the biological activity of lyophilized apricot extracts recovered from apricot pomace. Antioxidants.

[bib4] Chen Y., Yang J., Meng Q., Tong H. (2023). Non-volatile metabolites profiling analysis reveals the tea flavor of “Zijuan” in different tea plantations. Food Chem..

[bib5] Contreras-Lopez E., Castañeda-Ovando A., González-Olivares L.G., Añorve-Morga J., Jaimez-Ordaz J. (2014). Effect of light on stability of anthocyanins in ethanolic extracts of rubus fruticosus. Food Nutr. Sci..

[bib6] Corrigan H., Dunne A., Purcell N., Guo Y., Wang K., Xuan H., Granato D. (2023). Conceptual functional-by-design optimisation of the antioxidant capacity of trans-resveratrol, quercetin, and chlorogenic acid: application in a functional tea. Food Chem..

[bib7] de Moura C., Kabbas Junior T., Mendanha Cruz T., Boscacci Marques M., Araújo Vieira do Carmo M., Turnes Pasini Deolindo C., Daguer H., Azevedo L., Xu Y.Q., Granato D. (2023). Sustainable and effective approach to recover antioxidant compounds from purple tea (Camellia sinensis var. assamica cv. Zijuan) leaves. Food Res. Int..

[bib8] de Moura C., Kabbas Junior T., Pedreira F.R. de O., Azevedo L., Furtado M.M., Sant'Ana A.S., Franchin M., Gonzaga V.R., Cui Y., Wen M., Zhang L., Pereira R.P., Granato D. (2022). Purple tea (Camellia sinensis var. assamica) leaves as a potential functional ingredient: from extraction of phenolic compounds to cell-based antioxidant/biological activities. Food Chem. Toxicol..

[bib9] do Carmo M.A.V., Granato D., Azevedo L. (2021). Advances in Food and Nutrition Research (1st ed., Vol. 98).

[bib10] do Carmo M.A.V., Pressete C.G., Marques M.J., Granato D., Azevedo L. (2018). Polyphenols as potential antiproliferative agents: scientific trends. Curr. Opin. Food Sci..

[bib11] Enaru B., Drețcanu G., Pop T.D., Stǎnilǎ A., Diaconeasa Z. (2021). Anthocyanins: factors affecting their stability and degradation. Antioxidants.

[bib12] Escher G.B., Wen M., Zhang L., Rosso N.D., Granato D. (2020). Phenolic composition by UHPLC-Q-TOF-MS/MS and stability of anthocyanins from Clitoria ternatea L. (butterfly pea) blue petals. Food Chem..

[bib13] Fan J.P., Fan C., Dong W.M., Gao B., Yuan W., Gong J.S. (2013). Free radical scavenging and anti-oxidative activities of an ethanol-soluble pigment extract prepared from fermented Zijuan Pu-erh tea. Food Chem. Toxicol..

[bib14] Gao X., Ho C.T., Li X., Lin X., Zhang Y., Chen Z., Li B. (2018). Phytochemicals, anti-inflammatory, antiproliferative, and methylglyoxal trapping properties of zijuan tea. J. Food Sci..

[bib15] Gonçalves Bortolini D., Windson Isidoro Haminiuk C., Cristina Pedro A., de Andrade Arruda Fernandes I., Maria Maciel G. (2021). Processing, chemical signature and food industry applications of Camellia sinensis teas: an overview. Food Chem. X.

[bib16] Granato D., Grevink R., Zielinski A.A.F., Nunes D.S., Van Ruth S.M. (2014). Analytical strategy coupled with response surface methodology to maximize the extraction of antioxidants from ternary mixtures of green, yellow, and red teas (camellia sinensis var. sinensis). J. Agric. Food Chem..

[bib17] Granato D., Reshamwala D., Korpinen R., Azevedo L., Vieira do Carmo M.A., Cruz T.M., Marques M.B., Wen M., Zhang L., Marjomäki V., Kilpeläinen P. (2022). From the forest to the plate – hemicelluloses, galactoglucomannan, glucuronoxylan, and phenolic-rich extracts from unconventional sources as functional food ingredients. Food Chem..

[bib18] Granato D., Santos J.S., Salem R.D., Mortazavian A.M., Rocha R.S., Cruz A.G. (2018). Effects of herbal extracts on quality traits of yogurts, cheeses, fermented milks, and ice creams: a technological perspective. Curr. Opin. Food Sci..

[bib19] Kerio L.C., Wachira F.N., Wanyoko J.K., Rotich M.K. (2012). Characterization of anthocyanins in Kenyan teas: extraction and identification. Food Chem..

[bib20] Kerio L.C., Wachira F.N., Wanyoko J.K., Rotich M.K. (2013). Total polyphenols, catechin profiles and antioxidant activity of tea products from purple leaf coloured tea cultivars. Food Chem..

[bib21] Kilel E.C., Faraj A.K., Wanyoko J.K., Wachira F.N., Mwingirwa V. (2013). Green tea from purple leaf coloured tea clones in Kenya - their quality characteristics. Food Chem..

[bib22] Khoo H.E., Azlan A., Tang S.T., Lim S.M. (2017). Anthocyanidins and anthocyanins: colored pigments as food, pharmaceutical ingredients, and the potential health benefits. Food Nutr. Res..

[bib23] Lees D.H., Francis F.J. (1972). Standardization of pigment analyses in cranberries. Hortscience.

[bib24] Li X.-X., Li Z.-Y., Zhu W., Wang Y.Q., Liang Y.R., Wang K.R., Ye J.H., Lu J.L., Zheng X.Q. (2023). Anthocyanin metabolism and its differential regulation in purple tea (Camellia sinensis). Plant Physiol. Biochem..

[bib25] Li Y., Cheng Z., Wang K., Zhu X., Ali Y., Shu W., Bao X., Zhu L., Fan X., Murray M., Zhou F. (2021). Procyanidin B2 and rutin in Ginkgo biloba extracts protect human retinal pigment epithelial (RPE) cells from oxidative stress by modulating Nrf2 and Erk1/2 signalling. Exp. Eye Res..

[bib26] Lv H.P., Dai W.D., Tan J.F., Guo L., Zhu Y., Lin Z. (2015). Identification of the anthocyanins from the purple leaf coloured tea cultivar Zijuan (Camellia sinensis var. assamica) and characterization of their antioxidant activities. J. Funct.Foods.

[bib27] Margraf T., Santos É.N.T., de Andrade E.F., van Ruth S.M., Granato D. (2016). Effects of geographical origin, variety and farming system on the chemical markers and in vitro antioxidant capacity of Brazilian purple grape juices. Food Res. Int..

[bib28] Migliorini A.A., Piroski C.S., Daniel T.G., Cruz T.M., Escher G.B., Vieira do Carmo M.A., Azevedo L., Marques M.B., Granato D., Rosso N.D. (2019). Red chicory (cichorium intybus) extract rich in anthocyanins: chemical stability, antioxidant activity, and antiproliferative activity in vitro. J. Food Sci..

[bib29] Mu H., Zhang A., Zhang W., Cui G., Wang S., Duan J. (2012). Antioxidative properties of crude polysaccharides from Inonotus obliquus. Int. J. Mol. Sci..

[bib30] Pedro A.C., Granato D., Rosso N.D. (2016). Extraction of anthocyanins and polyphenols from black rice (Oryza sativa L.) by modeling and assessing their reversibility and stability. Food Chem..

[bib31] Pedro A.C., Moreira F., Granato D., Rosso N.D. (2016). Extraction of bioactive compounds and free radical scavenging activity of purple basil (Ocimum basilicum L.) leaf extracts as affected by temperature and time. Annals Brazilian Academy Sci..

[bib32] Rajasekar M., Bhuvanesh P., Varada P., Selvam M. (2023). Results in Chemistry Review on anticancer activity of flavonoid derivatives : recent developments and future perspectives. Res. Chem..

[bib33] Santos J.S., Alvarenga Brizola V.R., Granato D. (2017). High-throughput assay comparison and standardization for metal chelating capacity screening: a proposal and application. Food Chem..

[bib34] Santos J.S., Deolindo C.T.P., Hoffmann J.F., Chaves F.C., do Prado-Silva L., Sant'Ana A.S., Azevedo L., do Carmo M.A.V., Granato D. (2018). Optimized Camellia sinensis var. sinensis, Ilex paraguariensis, and Aspalathus linearis blend presents high antioxidant and antiproliferative activities in a beverage model. Food Chem..

[bib35] Shen J., Zou Z., Zhang X., Zhou L., Wang Y., Fang W., Zhu X. (2018). Metabolic analyses reveal different mechanisms of leaf color change in two purple-leaf tea plant (Camellia sinensis L.) cultivars. Horticult. Res..

[bib36] Shi J., Simal-Gandara J., Mei J., Ma W., Peng Q., Shi Y., Xu Q., Lin Z., Lv H. (2021). Insight into the pigmented anthocyanins and the major potential co-pigmented flavonoids in purple-coloured leaf teas. Food Chem..

[bib37] Suganya K., Poornima A., Sumathi S., Chigurupati S., Alyamani N.M., Ghazi Felemban S., Bhatia S., Al-Harrasi A., Sayed Moawad A. (2022). Rutin induces endoplasmic reticulum stress-associated apoptosis in human triple-negative breast carcinoma MDA-MB-231 cells – in vitro and in silico docking studies. Arab. J. Chem..

[bib38] Tomas M., Garcia-Perez P., Rivera-Perez A., Patrone V., Giuberti G., Lucini L., Capanoglu E. (2024). The addition of polysaccharide gums to *Aronia melanocarpa* purees modulates the bioaccessibility of phenolic compounds and gut microbiota: a multiomics data fusion approach following in vitro digestion and fermentation. Food Chem..

[bib39] Xu Y.Q., Zhang Y.N., Chen J.X., Wang F., Du Q.Z., Yin J.F. (2018). Quantitative analyses of the bitterness and astringency of catechins from green tea. Food Chem..

[bib40] Zenin V., Ivanova J., Pugovkina N., Shatrova A., Aksenov N., Tyuryaeva I., Kirpichnikova K., Kuneev I., Zhuravlev A., Osyaeva E., Lyublinskaya E., Gazizova I., Guriev N., Lyublinskaya O. (2022). Resistance to H_2_O_2_-induced oxidative stress in human cells of different phenotypes. Redox Biol..

[bib41] Zhao Y., Lai W., Xu A., Jin J., Wang Y., Xu P. (2020). Characterizing relationships among chemicals, sensory attributes and in vitro bioactivities of black tea made from an anthocyanins-enriched tea cultivar. LWT.

[bib42] Zhang L., Santos J.S., Cruz T.M., Marques M.B., do Carmo M.A.V., Azevedo L., Wang Y., Granato D. (2019). Multivariate effects of Chinese keemun black tea grades (Camellia sinensis var. sinensis) on the phenolic composition, antioxidant, antihemolytic and cytotoxic/cytoprotection activities. Food Res. Int..

