# Supplementary material for: Anthocyanin-rich extract from purple tea: Chemical stability, cellular antioxidant activity, and protection of human erythrocytes and plasma
Source: Curr Res Food Sci. 2024 Feb 16;8:100701. doi: 10.1016/j.crfs.2024.100701 (PMC10906145; doi:10.1016/j.crfs.2024.100701)
Supplement: Multimedia component 1 [file mmc1.docx]

**Supplementary Material**

**Table 1**: Regression equations, retention times for the compounds quantified by LC-MS, determination coefficients (R^2^), the lower limit of quantification (LLOQ), and the upper limit of quantification (ULOQ).

| **Compound** | **RT (min)** | **Regression Equation** | **R^2^** | **LLOQ** | **ULOQ** |
| --- | --- | --- | --- | --- | --- |
| Cyanidin-3,5-*O*-diglucoside | 5.37 | y = 8.61288e4 x - 12945.10909 | 0.99897 | 5 | 5000 |
| Cyanidin-3-(6-*O*-*p*-caffeoyl)-glucoside | 11.39 | y = 3.62073e4 x + 4600.17059 | 0.99612 | N/A | N/A |
| Cyanidin-3-*O*-glucoside | 7.4 | y = 1.32271e5 x + 27881.98074 | 0.99872 | 5 | 5000 |
| Delphinidin-3-*O*-(6-*O*-*p*-coumaroyl)-glucoside | 11.92 | y = 3.62073e4 x + 4600.17059 | 0.99612 | N/A | N/A |
| Delphinidin-3-*O*-5-*O*-(6-*O*-coumaroyl)-diglucoside | 10.83 | y = 3.62073e4 x + 4600.17059 | 0.99612 | N/A | N/A |
| Delphinidin-3,5-*O*-diglucoside | 4.42 | y = 3.62073e4 x + 4600.17059 | 0.99612 | 10 | 5000 |
| Pelargonidin-3-*O*-galactoside | 7.73 | y = 3.62073e4 x + 4600.17059 | 0.99612 | N/A | N/A |
| Peonidin-3-*O*-(6-*O*-*p*-coumaroyl)-glucoside | 12.64 | y = 3.62073e4 x + 4600.17059 | 0.99612 | N/A | N/A |
| Peonidin-3-*O*-galactoside | 8.42 | y = 3.62073e4 x + 4600.17059 | 0.99612 | N/A | N/A |
| Petunidin-3-*O*-arabinoside | 8.62 | y = 3.62073e4 x + 4600.17059 | 0.99612 | N/A | N/A |
| Procyanidin A2 | 9.15 | y = 15286.76550 x + 8133.51773 | 0.99727 | 5 | 5000 |
| Procyanidin C1 | 6.74 | y = 879.61137 x + 778.01215 | 0.99241 | 20 | 5000 |
| Procyanidin B3 | 3.52 | y = 5461.82105 x + 755.95270 | 0.99416 | 2 | 5000 |
| Procyanidin B2 | 5.5 | y = 7118.22554 x - 549.63337 | 0.99882 | 5 | 5000 |
| Procyanidin B1 | 3.9 | y = 9097.67668 x + 1351.46847 | 0.99611 | 2 | 5000 |
| Procyanidin A1 | 7.1 | y = 3.90387e4 x + 9811.51856 | 0.99632 | 2 | 5000 |
| Naringenin | 13.05 | y = 4.38422e4 x + 21202.58917 | 0.99900 | 5 | 5000 |
| Naringenin-7-*O*-glucoside | 11.33 | y = 6052.14483 x + 13442.20102 | 0.99931 | 20 | 5000 |
| Afzelin | 12.84 | y = 2389.49390 x + 1796.00699 | 0.99889 | 20 | 5000 |
| Dihydrokaempferol | 10.72 | y = 14469.86897 x + 4.07541e4 | 0.99456 | 50 | 5000 |
| Quercetin-3-*O*-glucoside | 11.24 | y = 6183.51163 x + 21920.53894 | 0.99850 | 50 | 5000 |
| Kaempferol-3-*O*-rutinoside | 12.41 | y = 1253.54691 x + 1466.65486 | 0.99907 | 5 | 5000 |
| Rutin | 11.26 | y = 3167.10737 x + 2768.42378 | 0.99817 | 10 | 5000 |
| Dihydromyricetin | 5.64 | y = 2679.97734 x + 1010.99332 | 0.99582 | 20 | 5000 |

Note: N/A: not applicable.


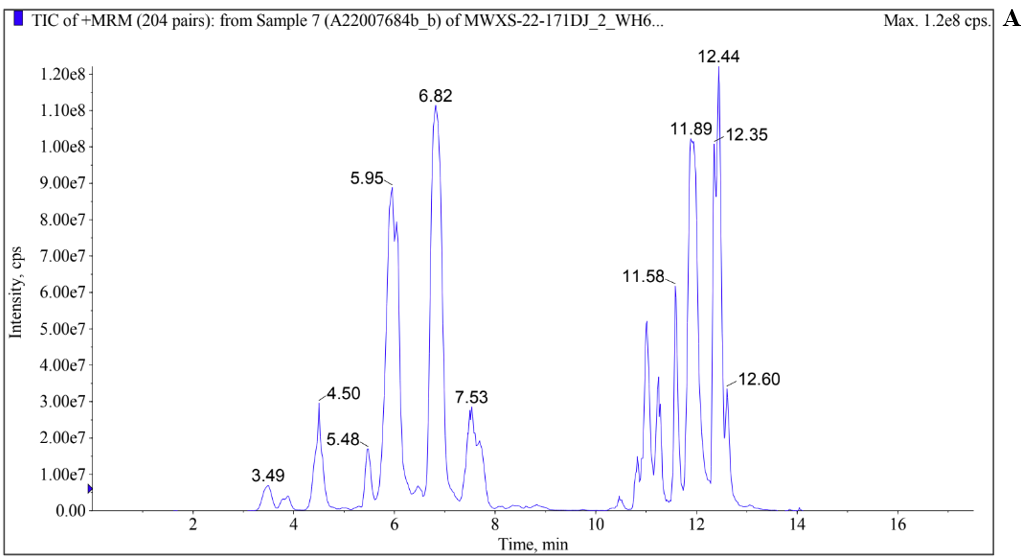


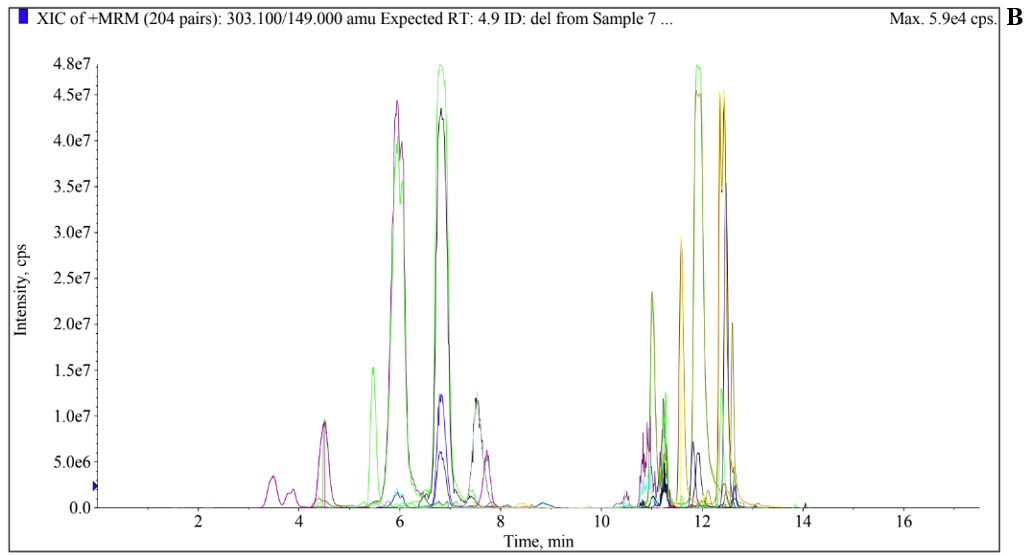


**
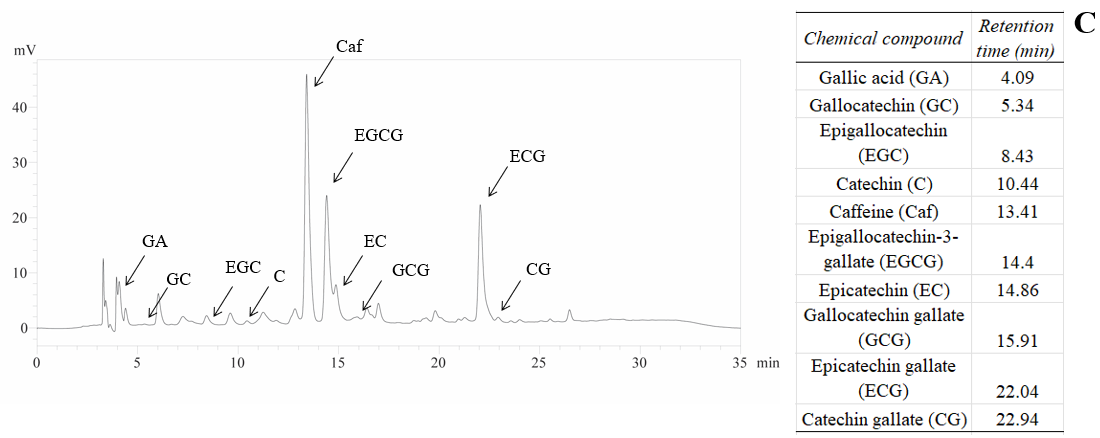
**

**Figure 1**: LC-MS chromatogram of total ions (A) and extracted ions (B) for analysing phenolic compounds in the lyophilized purple tea extract. HPLC chromatogram of lyophilized purple tea extract.
